# Editorial: Advances in β-cell development & regeneration

**DOI:** 10.3389/fendo.2026.1765933

**Published:** 2026-01-15

**Authors:** Santiago A. Rodríguez-Seguí, Meritxell Rovira, George K. Gittes

**Affiliations:** 1 Instituto de Fisiología, Biología Molecular y Neurociencias (IFIBYNE-UBA-CONICET), Universidad de Buenos Aires, Facultad de Ciencias Exactas y Naturales, Buenos Aires, Argentina; 2 Departamento de Fisiología, Biología Molecular y Celular, Universidad de Buenos Aires, Facultad de Ciencias Exactas y Naturales, Ciudad Universitaria, Buenos Aires, Argentina; 3 Department of Physiological Science, School of Medicine, University of Barcelona (UB), L’Hospitalet de Llobregat, Barcelona, Spain; 4 Pancreas Regeneration Group, Oncobell Program, Institut D’Investigació Biomèdica de Bellvitge (IDIBELL), L’Hospitalet de Llobregat, Barcelona, Spain; 5 CIBER de Diabetes y Enfermedades Metabólicas Asociadas (CIBERDEM), Instituto de Salud Carlos III, Barcelona, Spain; 6 Division of Pediatric Surgery, University of Pittsburgh Medical Center (UPMC) Children’s Hospital of Pittsburgh, Pittsburgh, PA, United States

**Keywords:** beta cell, development, diabetes, differentiation, modeling, niche, pancreas, regeneration

The pathogenesis of diabetes mellitus is fundamentally linked to a decline in functional pancreatic β-cell mass (1). Replacing these insulin-producing cells remains a central goal of regenerative medicine and is pursued through two primary avenues: guiding the differentiation of human pluripotent stem cells *in vitro* and harnessing the innate regenerative capacity of the pancreas *in vivo* (2, 3). Both strategies are deeply informed by developmental cues and molecular mechanisms that govern β-cell development, differentiation, and identity (4–6). This Research Topic compiles a series of articles that advance our understanding of the key drivers of β-cell formation, function, and regeneration. These contributions collectively push the frontiers of regenerative medicine for diabetes, especially cell replacement therapies.

Delving into the molecular machinery, several articles illuminate key pathways that govern β-cell fate and adaptive plasticity. Two studies highlight critical regulators of β-cell mass. D’Addio et al. identify the TMEM219/IGFBP3 axis as a novel checkpoint in β-cell precursors. They demonstrate that activation of TMEM219 by its ligand, IGFBP3, induces caspase-8-mediated cell death, while its inhibition—pharmacologically or via the key regulator miR-129-2—promotes precursor proliferation. This pathway is a promising target for unlocking the regenerative potential of endogenous precursors in type 1 diabetes (T1D). Complementarily, de Ramos et al. provide *in vivo* evidence for the essential role of the transcription factor HNF4α in the β-cell’s adaptive response to metabolic stress. In a high-fat diet model, β-cell-specific HNF4α knockout prevented compensatory β-cell mass expansion, exacerbating glucose intolerance and underscoring its non-redundant role in maintaining functional mass.

Further exploring this layer of transcriptional regulation, Wong and Alejandro present a mini-review on how post-translational modifications (PTMs) fine-tune key transcription factors, such as Pdx1 and Nkx6.1. They elaborate on how nutrients and stress signals, through PTMs such as phosphorylation and O-GlcNAcylation, dynamically control β-cell identity, function, and survival, adding a crucial layer of complexity to our understanding of β-cell homeostasis.

Translating this basic knowledge into human-specific models is critical for therapeutic advancement. The review by Ka et al. presents the use of advanced *ex vivo* models, such as induced pluripotent stem cells (iPSCs) and pancreatic organoids, to study Maturity-Onset Diabetes of the Young (MODY). These patient-derived systems bridge the gap between rodent models and human pathophysiology, offering powerful platforms for dissecting disease mechanisms, advancing drug discovery, and developing autologous cell therapy.

These molecular and cellular insights are directly relevant to the clinical context of Type 2 Diabetes (T2D). The original research by Cheng et al. analyzes data from nearly 3,000 individuals to propose a “three-phase model” of β-cell function in T2D: an initial brief ascent post-diagnosis, a prolonged period of exponential decline, and a final stabilization at a low plateau. This model provides a crucial clinical framework by identifying modifiable factors, such as BMI, that influence this trajectory. In contrast, the mini-review by Vasavada and Dhawan synthesizes molecular strategies to counteract this decline through regeneration. The authors detail barriers to human β-cell replication—such as DYRK1A signaling—and explore promising therapeutic agents, such as harmine and denosumab, that can promote β-cell expansion in preclinical models, highlighting the need for combination therapies that also enhance cell survival.

This Research Topic concludes by revisiting a fundamental question: what is the true origin of new β-cells? Heidenreich et al. contribute a pivotal, data-driven perspective to this ongoing debate. Through a meticulous re-analysis of single-cell datasets, they report that the transcriptional signature of a previously described adult Procr+ progenitor population is not found in standard adult islet preparations but is instead identified in a subset of mesothelial cells in the embryonic pancreas of both mice and humans. Their work further identifies transcriptomic and epigenomic connections between these embryonic cells and ductal/endocrine progenitors. Notably, a PROCR-like cell subcluster was also found to be spontaneously co-specified during the differentiation of human induced pluripotent stem cells (iPSCs) into pancreatic progenitor cells. These compelling findings encourage a reinterpretation of the origin of Procr+ progenitor cells, suggesting a potential developmental link to the embryonic mesothelium and highlighting the critical importance of cellular context in regenerative biology.

## Future directions and concluding perspectives

The collective works in this Research Topic underscore key challenges for translational progress. A priority is ensuring the long-term stability and function of new β-cells, which must fully integrate into physiological glucose regulation without loss of identity or viability. This demands rigorous, long-term validation in disease-relevant models.

Furthermore, the field must decisively bridge the gap between rodent models and human biology (7, 8). Advancing human model systems—such as complex organoids and humanized mouse models—alongside the integration of human genomic data, will be essential to prioritizing clinically relevant targets (9–12).

From a therapeutic standpoint, achieving safe and targeted delivery of regenerative cues, whether pharmacological or genetic, is paramount. Strategies must minimize off-target effects, potentially leveraging emerging delivery technologies. Furthermore, the success of these approaches, especially in T1D, will hinge on modulating the pancreatic niche to create a supportive microenvironment that mimics *in vivo* physiological settings for new β-cells.

In conclusion, this Research Topic highlights the multifaceted path toward β-cell regeneration. By deepening our understanding of developmental principles, cellular plasticity, and niche interactions, the prospect of a curative therapy for diabetes continues to gain momentum.
